# High‐intensity interval training changes mitochondrial respiratory capacity differently in adipose tissue and skeletal muscle

**DOI:** 10.14814/phy2.13857

**Published:** 2018-09-17

**Authors:** Tine L. Dohlmann, Morten Hindsø, Flemming Dela, Jørn W. Helge, Steen Larsen

**Affiliations:** ^1^ Xlab Center for Healthy Aging Department of Biomedical Sciences Faculty of Health Sciences University of Copenhagen Copenhagen Denmark; ^2^ Department of Geriatrics Bispebjerg University Hospital Copenhagen Denmark; ^3^ Clinical Research Centre Medical University of Bialystok Bialystok Poland

**Keywords:** Adipose tissue, ADP sensitivity, high‐intensity training, mitochondria, mitochondrial respiratory capacity, skeletal muscle

## Abstract

The effect of high‐intensity training (HIT) on mitochondrial ADP sensitivity and respiratory capacity was investigated in human skeletal muscle and subcutaneous adipose tissue (SAT). Twelve men and women underwent 6 weeks of HIT (7 × 1 min at app. 100% of maximal oxygen uptake (VO_2max_)). Mitochondrial respiration was measured in permeabilized muscle fibers and in abdominal SAT. Mitochondrial ADP sensitivity was determined using Michaelis Menten enzyme kinetics. VO_2max_, body composition and citrate synthase (CS) activity (skeletal muscle) and mtDNA (SAT) were measured before and after training. VO_2max_ increased from 2.6 ± 0.2 to 2.8 ± 0.2 L O_2_/min (*P* = 0.011) accompanied by a decreased mitochondrial ADP sensitivity in skeletal muscle (*K*_m_: 0.14 ± 0.02 to 0.29 ± 0.03 mmol/L ADP (*P* = 0.002)), with no changes in SAT (*K*_m_: 0.12 ± 0.02 to 0.16 ± 0.05 mmol/L ADP;* P* = 0.186), following training. Mitochondrial respiratory capacity increased in skeletal muscle from 57 ± 4 to 67 ± 4 pmol O_2_·mg^−1^·sec^−1^ (*P* < 0.001), but decreased with training in SAT from 1.3 ± 0.1 to 1.0 ± 0.1 pmol O_2_·mg^−1^·sec^−1^ (*P* < 0.001). CS activity increased (*P* = 0.027) and mtDNA was unchanged following training. Intrinsic mitochondrial respiratory capacity was unchanged in skeletal muscle, but increased in SAT after HIT. In summary, our results demonstrate that mitochondrial adaptations to HIT in skeletal muscle are comparable to adaptations to endurance training, with an increased mitochondrial respiratory capacity and CS activity. However, mitochondria in SAT adapts differently compared to skeletal muscle mitochondria, where mitochondrial respiratory capacity decreased and mtDNA remained unchanged after HIT.

## Introduction

In the last decade our interest and knowledge regarding high‐intensity interval training has been revitalized. Regular endurance training and high‐intensity interval training (HIT; HIIT; SIT; REHIT; defined in the method section) increases maximal oxygen uptake (Tabata et al. 1996; Burgomaster et al. 2008; Hood et al. 2011; Ziemann et al. 2011; Tjonna et al. 2013; Larsen et al. 2015; Ruffino et al. 2017). This increase is accompanied by an increased mitochondria mass in the skeletal muscle (Burgomaster et al. 2008; Hood et al. 2011; Jacobs et al. 2013; Larsen et al. 2015). However, the literature is sparse in regard to the effect of training on mitochondrial intrinsic respiratory capacity. In studies of high‐intensity training (HIT) (Jacobs et al. 2013; Larsen et al. 2015; Granata et al. 2016) and of a combination of endurance training and HIT (Pesta et al. 2011), mitochondrial respiratory capacity was increased, but intrinsic mitochondrial respiratory capacity (mitochondrial respiratory capacity normalized to mitochondrial content (CS activity)) appears to remain unchanged (Jacobs et al. 2013; Larsen et al. 2015). Granata and colleagues (2016) measured mitochondrial respiratory capacity and used CS activity as a biomarker for mitochondrial function, and found an increased intrinsic mitochondrial respiratory capacity following 4 weeks of sprint interval training, but unchanged following high‐intensity interval training and regular endurance training (Granata et al. 2016). Lundby and colleagues recently reported that 6 weeks of endurance training decreases intrinsic mitochondrial respiratory capacity when normalized to mitochondrial volume density (Meinild Lundby et al. 2018). Indicating that the adaptations in the mitochondria could be different between training modalities.

Chance and Williams postulated in the 1950s that the mitochondrial oxidative phosphorylation system was controlled by ADP (Chance and Williams 1955). The literature regarding mitochondrial ADP sensitivity is sparse. It has been reported that ADP sensitivity in skeletal muscle decreases along with an increased VO_2max,_ following a combination of endurance and interval training (Walsh et al. 2001a), which suggests an inverse relationship (ADP sensitivity was measured in the presence of malate and pyruvate). This is supported by cross‐sectional data, where the most athletic of three groups (the group with the highest VO_2max_), had a higher apparent *K*_m_ for ADP (Zoll et al. 2002), in this study ADP sensitivity was measured in the presence of malate and glutamate. ADP sensitivity with malate and glutamate was also investigated after high‐intensity interval training, where an increased sensitivity was seen (Ydfors et al. 2016). Walsh and colleagues proposed that the decreased sensitivity for ADP could be due to a change toward a more oxidative muscle after a training intervention (Walsh et al. 2001a), whereas Perry and colleagues previously have linked decreased mitochondrial ADP sensitivity to improved phosphate‐transferring capacity by creatine kinase (CK) (Perry et al. 2012). It has been argued that phosphate transferring by creatine kinase (CK) is most predominant in oxidative muscle fibers (Sahlin and Harris 2011), which is in line with other findings (Walsh et al. 2001a; Perry et al. 2012). However, in glycolytic muscle fibers, phosphate transferring by the adenylate kinase (AK) system is most pronounced (Sahlin and Harris 2011), and as human muscles as the vastus lateralis investigated in this study have a heterogenous fiber type composition (approximately 42% oxidative and 58% glycolytic fibers (Staron et al. 2000)), it is possible that both CK and AK play a role.

Accumulation of white adipose tissue (including subcutaneous adipose tissue) has massive consequences for metabolic health. The ability to increase activity of white adipose tissue would have a great impact on metabolic health, this could potentially be done with physical activity. Few studies have previously investigated mitochondrial respiration in human adipose tissue (Hallgren et al. 1986; Kraunsoe et al. 2010), and to our knowledge only one study has investigated the effect of training on subcutaneous adipose tissue (sampled close to the umbilicus) (Larsen et al. 2015) where no changes were seen following training. Trained women have a decreased fat cell mass and a decreased adipose tissue area (sample obtained from the umbilical region and femoral site) (Mauriege et al. 1997), and it has been found that mitochondrial content does not change in human abdominal adipose tissue after a training period of 10 days (Camera et al. 2010).

We investigated the effects of 6 weeks of low volume HIT on mitochondrial ADP sensitivity, mitochondrial respiratory capacity, and mitochondrial content in skeletal muscle fibers and in subcutaneous adipose tissue in overweight sedentary adults, to see if skeletal muscle and adipose tissue adapts to 6 weeks of HIT, or adipose tissue need a longer exposure. We hypothesized that mitochondrial respiratory capacity would increase, and that mitochondrial ADP sensitivity would decrease in skeletal muscle with no changes in adipose tissue after HIT.

## Materials and Methods

### Subjects

Twelve healthy sedentary subjects (5 males, 7 females) aged 40 ± 2 years (range 24–50 years) with a body mass index (BMI) of 32 ± 2 kg·m^−2^ were included in this study. Subjects’ characteristics are given in Table 1. The subjects habitual activity level was evaluated by the International Physical Activity Questionnaire (IPAQ) (Craig et al. 2003), where subjects with a weekly score of <600 MET‐minutes were considered inactive and included in the study. Subjects were not taking any medication, but the use of pharmacological contraception was allowed. The subjects were informed of the nature and possible risks associated with the study, before they volunteered and gave written consent to participate. The study was approved by Copenhagen Ethics Committee (journal nr H‐3‐2012‐024), and adhered to the Principles of the Declaration of Helsinki. The samples for this project are part of a larger EU funded project “Metapredict” 7th Framework.

**Table 1:** Subject characteristics

	Pre‐HIT	Post‐HIT	*P*‐value
Age (years)	40 ± 2	–	
BMI (kg·m^−2^)	32 ± 2	32 ± 1	0.529
LBM (kg)	55 ± 4	55 ± 4	0.304
BW (kg)	98 ± 6	99 ± 6	0.435
FAT (%)	41 ± 2	41 ± 2	0.321
Fasting glucose (mmol/L)	4.6 ± 0.1	4.6 ± 0.2	0.827
Fasting insulin (pmol/L)	64 ± 9	70 ± 9	0.408
VO_2max_ (L O_2_·min^−1^)	2.6 ± 0.2	2.8 ± 0.2	0.011
VO_2max_ (mL O_2_·min^−1^·kg^−1^)	27 ± 2	29 ± 2	0.010
TTF (min)	13.8 ± 1.7	15.4 ± 1.7	0.003
Load_max_ (Watt)	210 ± 16	230 ± 17	<0.001

Values are means ± SEM. *N* = 12 (5M/7F). Abbreviations: BMI, body mass index; LBM, lean body mass; BW, body weight; FAT%, body fat percentage; VO_2max_, maximal oxygen consumption; Load_max_, maximal load achieved during the maximal oxygen consumption test; TTF, time to fatigue.

### Experimental design

Prior to the training period subjects underwent a day of testing (PRE). Subjects came fasted to the laboratory in the morning (8–9 am). A dual X‐ray absorptiometry (DXA) scan (Lunar iDXA, GE medical Systems Lunar, Madison, Wisconsin, USA) was performed to determine body composition. Biopsies were obtained from m. vastus lateralis and from subcutaneous abdominal adipose tissue (taken app. 5 cm from the umbilicus) under local anesthesia (5% Lidocaine) using the Bergström needle modified for suction. A portion of the muscle and adipose tissue was kept in the relaxing medium “BIOPS” (10 mmol/L Ca‐EGTA buffer, 0.1 *μ*mol/L free calcium, 20 mmol/L imidazole, 20 mmol/L taurine, 50 mmol/L K‐MES, 0.5 mmol/L DTT, 6.56 mmol/L MgCl_2_, 5.77 mmol/L ATP, 15 mmol/L creatine phosphate, pH 7.1, at 0°C) for immediately analysis of mitochondrial respiratory capacity using high‐resolution respirometry, and samples for enzyme analysis (skeletal muscle) and mtDNA quantification (SAT) were snap frozen in liquid nitrogen within 30 sec after sampling and stored at −80°C.

After inserting a catheter in a dorsal hand vein, a fasting blood sample was taken using the heated hand technique. Blood glucose was measured directly (YSI analyzer, 2300 STAT plus; Yellow Springs, Ohio, USA). Blood for insulin analyses was collected in tubes containing 25 *μ*L Trasylol Ethylenediaminetetra‐acetic acid (trEDTA), and immediately centrifuged at 2000*g* in 4°C for 10 min. The plasma was transferred to microtubes, and stored at −80°C until analyzed using a Dako A/S (Elektra‐Box Diagnostica ApS, Glostrup, Denmark) ELISA kit.

One hour after ingesting a meal (sandwich and juice) a resting electrocardiogram was performed to screen for any heart conditions before doing strenuous exercise. Subsequently subjects completed an incremental test to exhaustion on a Lode Corival stationary bike (Lode B.V., Groningen, the Netherlands). After a 2‐min warm up on the load corresponding to their body weight (1 W·kg bw^−1^), the load increased with 25 W·3 min^−1^ until voluntary exhaustion. Expired air was analyzed using COSMED online gas exchange system (COSMED, Rome, Italy). Achievement of VO_2max_ was accepted when a respiratory exchange ratio >1.20, maximal heart rate (220 – age) was present, and a leveling off or a decline in VO_2_ was reached. The duration of the test was recorded and called time to fatigue (TTF). After exhaustion, subjects rested for 5 min before they returned to the bike and cycled for 2 min on the load corresponding to body weight (1 W·kg bw^−1^), then 2 min on the maximal load achieved on the previous test, followed by increments of 0.42 W·sec^−1^ (ramp) until exhaustion. Heart rate was recorded continuously (Polar Electro T31, Polar, Kempele, Finland).

The VO_2max_ was repeated approximately 1 week after completing the first test day, and the highest VO_2max_ from the two test days was considered baseline VO_2max_. Overall five subjects reached the highest baseline VO_2max_ on the first test day, and seven subjects reached it on the second day. The differences in VO_2max_ between the two test days were on average 0.5 ± 1.7%. Three days following the last training session a final test (POST) day was completed, including muscle and fat biopsies and DXA.

### Training

The subjects completed 18 HIT sessions over 6 weeks (100% supervised). Each session consisted of 2‐min warm up at 25 W, followed by 7 bouts of 1 min high‐intensity exercise interspersed with one min rest periods. The training intensity was gradually increased during the first three training sessions (session 1: 60, 70, 80, 90, 95, 95, 95%; session 2: 70, 80, 90, 95, 95, 95, 95%: session 3: 80, 90, 95, 95, 95, 95, 95% of the load corresponding to VO_2max_ (198 ± 17 W)). From session 4 to 6 the load was 95% of the load corresponding to VO_2max_, from session 7 to 12 the load was increased to 100%, ending at 105% for session 13 to 18. The subjects were instructed not to eat or drink for one hour before and after each training session, but water was allowed ad libitum. We refer to this type of training as high‐intensity training (HIT), although we are aware that others are also defining interval training (10 × 4 min) as high‐intensity interval training (HIIT) (Perry et al. 2008). Furthermore sprint interval training (SIT; short bouts (4–6 × 30 sec with high load; Wingate test)) is also seen in the literature (Granata et al. 2016), as well as reduced exertion high‐intensity training (REHIT; short bouts (1–2 × 10–20 sec with high load; Wingate test) (Metcalfe et al. 2012).

### Analytical methods

Mitochondrial respiration was measured in saponin‐permeabilized muscle fibers (Pfi) (from all 12 subjects, 5 males and 7 females) and in digitonin treated adipose tissue (from 8 subjects; 5 males and 3 females). The permeabilization procedure was as previously described (Kuznetsov et al. 2008). All measurements were done using a high‐resolution respirometer (Oxygraph‐2k, Oroboros Instruments, Innsbruck, Austria). Prior to the experiments the Oxygraph was calibrated to correct for back‐diffusion of oxygen into the chamber, leak from the exterior, oxygen consumption by the chemical medium and by the polarographic oxygen sensor. O_2_ flux was resolved by software (Datlab 5, Oroboros Instruments, Innsbruck, Austria). All respirometry measurements were done in duplicate in the respiration medium MiR05 (110 mmol/L sucrose, 60 mmol/L potassium lactobionate, 0.5 mmol/L EGTA, 3 mmol/L MgCl_2_·6H_2_O, 20 mmol/L taurine, 10 mmol/L KH_2_PO_4_, 20 mmol/L HEPES, 1 g/L BSA, pH 7.1 at 37°C) at 37°C after hyperoxygenation (450–200 nmol·mL^−1^) to avoid oxygen limitations (hyperoxygenation was only used in the skeletal muscle measurements).

One titration protocol was similar for both skeletal muscle and adipose tissue, except that the titration protocol in adipose tissue was initiated by titration of 2 *μ*L digitonin (2 *μ*mol/L) to permeabilize the adipocytes. Malate (2 mmol/L) and glutamate (10 mmol/L) were added as NADH‐related substrates feeding electrons into complex I (LEAK, CI_*L*_). ADP was titrated in step‐wise increments (0.05–0.1–0.25–0.5–1.0–2.5–5.0 mmol/L). When the medium was saturated with ADP (5 mmol/L; CI_*P*_), succinate (10 mmol/L) was added as a FADH_2_‐related substrate feeding electrons into complex II (CI + II_*P*_). The adipose tissue weight in the respiratory chamber did not differ from pre to post intervention (34 ± 4 vs. 39 ± 3 mg). A second titration protocol was conducted in skeletal muscle. Malate (2 mmol/L; LEAK, CI_*L*_) was added followed by ADP (5 mmol/L; CI_*P*_), then glutamate was titrated in step‐wise increments (0.2–0.5–1.0–2.0–5.0–10.0–25.0–50.0 mmol/L; CI_*P*_), succinate (10 mmol/L; CI + II_*P*_) was added as a FADH_2_‐related substrate feeding electrons into complex II and finally the protonophore (Carbonyl cyanide‐*4*‐(trifluoromethoxy)phenylhydrazone (FCCP)) was titrated in (ETS). Respiratory flux ratios were calculated. Substrate control ratio (SCR) refers to the ratio of CI_*P*_/CI + II_*P*_, LEAK/phosphorylation control ratio refers to the ratio of CI_*L*_/CI_*P*_), and coupling control ratio (CI + II_*P*_/CI + II_*E*_). The different ratios give an idea about intrinsic changes in the mitochondria. Cytochrome c was added in the protocols to test the integrity of the mitochondrial preparation, no increase in respiration was seen after addition in any of the experiments indicating that the preparation was reliable.

Intrinsic mitochondrial respiratory capacity was calculated as mitochondrial respiratory capacity normalized to CS and HAD activity in skeletal muscle and mtDNA in adipose tissue. The rationale for using mtDNA as a marker of mitochondrial content in adipose tissue and not in skeletal muscle is because there is only one DNA copy number per cell in adipose tissue, but this can vary in skeletal muscle between individuals.

Citrate synthase (CS) activity and *β*‐hydroxy‐acyl‐CoA dehydrogenase (HAD) activity were measured as previously described (Gram et al. 2014; Vigelso et al. 2016). Enzyme activities are expressed as micromoles substrate per minute per gram dw of muscle tissue.

Skeletal muscle creatine kinase (CK) was quantified on a Hitachi Cobas 6000 chemistry analyzer (Roche A/S, Hvidovre, Denmark) using a commercial available kit (Roche A/S, Hvidovre, Denmark).

Quantification of mtDNA was done in adipose tissue to estimate mitochondrial density, as previously described (Kraunsoe et al. 2010). Briefly levels of mtDNA and gDNA were determined by real‐time PCR (MX3005P QPCR machine; Stratagene, La Jolla, CA, USA). The concentration of mtDNA per milligram of tissue was used as an estimate of the amount of mitochondria per milligram tissue. Every nucleus contains two double stranded gDNA molecules of each chromosome, and adipocytes only have one nucleus per cell, the number of cells can be calculated (double stranded gDNA (per milligram of tissue)/2). The total number of adipocytes is calculated from both the adipocyte and non adipocyte fraction.

### Statistics and calculations

Data are presented as means ± SEM. Significance was set at *P *< 0.05. Due to a modest sample size there was not enough statistical power to consider gender differences, thus the subjects were considered as one group. Statistical analyses were performed using Student's paired *t* test and two‐way ANOVA for repeated measures as appropriate. Correlations were performed with Pearsons test.

The respiration rate before any ADP was added (CI_*L*_) was subtracted from the respiration at each ADP increment, to measure mitochondrial ADP sensitivity (Larsen et al. 2011). Statistics were done using SigmaPlot 13 (Systat software inc., Erkrath, Germany), and graphs were done with GraphPad Prism 7.0 (1992–2012 GraphPad Software, Inc.)

## Results

Following HIT, BMI, LBM, and body fat percentage were unchanged (Table 1). VO_2max_ increased 9% (*P* = 0.011), time to fatigue (TTF) increased 12% (*P* = 0.003), and maximal workload during the VO_2max_ test (Load_max_), increased with training (*P* < 0.001) (Table 1). Fasting glucose and insulin was unchanged following training (Table 1).

### Adaptations in adipose tissue

**Figure 1:**
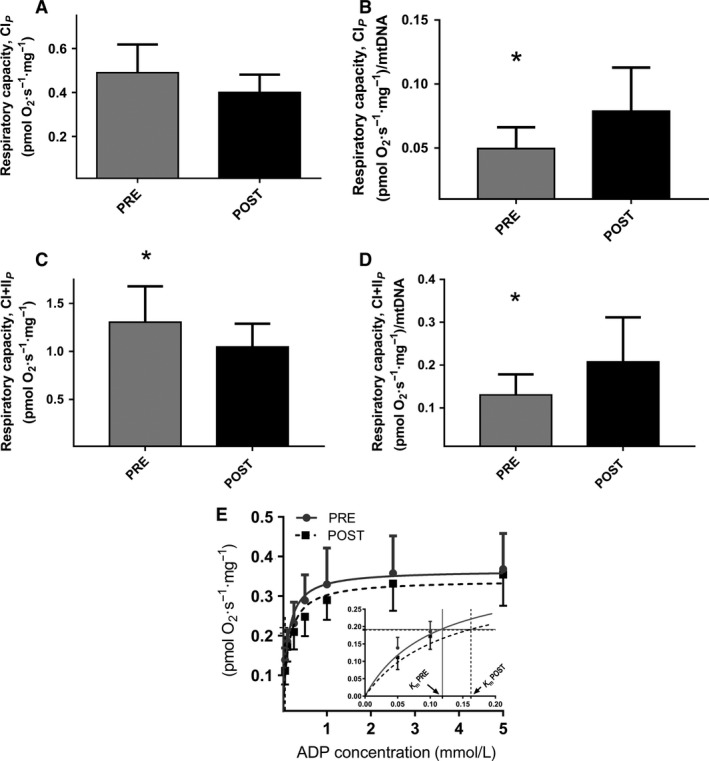
Mitochondrial respiratory capacity in subcutaneous adipose tissue. (A) with complex I linked substrates (CI_*P*_) (mass‐specific respiratory capacity) (*n* = 8; 3 females and 5 males). (B) Intrinsic mitochondrial respiratory capacity with complex I linked substrates (CI_*P*_) (mass‐specific respiratory capacity normalized to mtDNA) (*n* = 6; 2 females and 4 males). (C) with complex I + II linked substrates (CI + II_*P*_) (mass‐specific respiratory capacity) (*n* = 8; 3 females and 5 males). (D) Intrinsic mitochondrial respiratory capacity with complex I + II linked substrates (CI + II_*P*_) (mass‐specific respiratory capacity normalized to mtDNA) (*n* = 6; 2 females and 4 males). E) *K*_m_^app^ for ADP in subcutaneous adipose tissue, *n* = 8. Filled horizontal lines represent ½*V*_*max*_, and the corresponding vertical lines to the *x*‐axis are *K*_m_^app^ (ADP). The inserted figure illustrates ADP stimulated respiration and *K*_m_^app^ values on a scale from 0 to 0.2 mmol/L ADP. *Post‐HIT different from pre‐HIT,* P* < 0.05.

No changes were seen in mtDNA pr mg of tissue or mtDNA pr cell, but a tendency was seen for a decrease in cells pr mg tissue after the training intervention (Table 2). Mitochondrial respiratory capacity with complex I linked substrates (CI_*P*_) was unchanged after training (Fig. 1A). Maximal mitochondrial respiratory capacity (CI + II_*P*_) decreased 20% (Fig. 1C, *P* < 0.001) after training. Mitochondrial intrinsic respiratory capacity (respiration normalized to mtDNA) was increased following training (CI_*P*_ 59% (*P* = 0.016) and CI + II_*P*_ 60%, *n* = 6, *P *< 0.001, Fig. 1B and D). CI_*L*_/CI_*P*_ ratio was decreased following training with no change in SCR with succinate (Table 3). *V*_*max*_ and *K*_m_^app^ did not change significantly with training (0.37 ± 0.03 to 0.34 ± 0.03, *P* = 0.211, and 0.12 ± 0.02 to 0.16 ± 0.05 mmol/L, *P* = 0.186, respectively) (Fig. 1E). No difference was seen at baseline in ADP sensitivity from adipose tissue and skeletal muscle in the eight subjects analyzed (0.12 ± 0.02 to 0.14 ± 0.02 mmol/L, *P* = 0.320). A significant correlation was present between ADP sensitivity from skeletal muscle and adipose tissue before the intervention (*r* = 0.709; *P* = 0.05), but was not seen after the intervention (*r* = 0.253; *P* = 0.545).

**Table 2:** Skeletal muscle and subcutaneous adipose tissue analysis

	Pre‐HIT	Post‐HIT	*P*‐value
Skeletal muscle (*n* = 11)			
CS activity (*μ*mol·min^−1^·mg·dw^−1^)	119 ± 9	136 ± 11	0.027
HAD activity (*μ*mol·min^−1^·mg·dw^−1^)	103 ± 7	111 ± 11	0.236
Adipose tissue (*n* = 10)			
mtDNA (ds mtDNA·mg tissue^−1^·10^6^)	11 ± 2	7 ± 1	0.131
Cells per mg tissue	8026 ± 1272	5750 ± 1131	0.061
mtDNA per cell (ds mtDNA/(ds gDNA/2))	1373 ± 159	1430 ± 108	0.692

Data are means ± SEM. Abbreviations: CS, Citrate synthase; ds, Double stranded; HAD, *β*‐hydroxy‐acyl‐CoA dehydrogenase; mtDNA, Mitochondrial DNA; gDNA, Genomic DNA.

**Table 3:** Mitochondrial ratios in adipose tissue and skeletal muscle

	Pre‐HIT	Post‐HIT	*P*‐value
Adipose tissue			
CI_*L*_/CI_*P*_	0.24 ± 0.03	0.12 ± 0.02	0.034
CI_*P*_/CI + II_*P*_	0.38 ± 0.01	0.39 ± 0.02	0.406
Skeletal muscle			
CI_*L*_/CI_*P*_	0.13 ± 0.01	0.23 ± 0.03	0.004
CI_*P*_/CI + II_*P*_	0.45 ± 0.02	0.43 ± 0.02	0.312
CI + II_*P*_/CI + II_*E*_	0.67 ± 0.03	0.64 ± 0.03	0.274

Data are means ± SEM. CI_*L*_/CI_*P*_ and CI_*P*_/CI + II_*P*_ is calculated from the protocol applied on both adipose tissue and skeletal muscle (ADP sensitivity). CI + II_*P*_/CI + II_*E*_ was calculated from the protocol applied only on skeletal muscle (substrate sensitivity for glutamate).

### Adaptations in skeletal muscle

**Figure 2:**
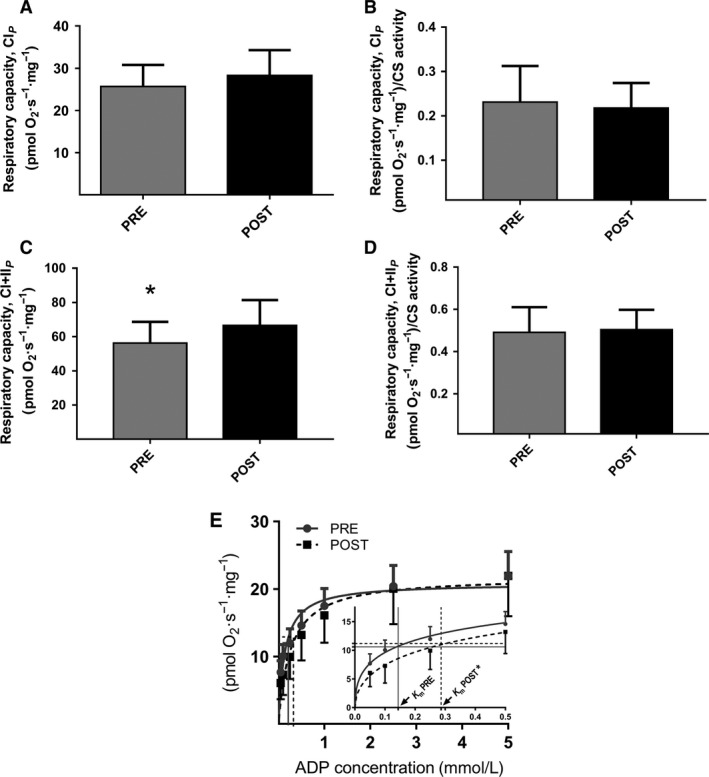
Mitochondrial respiratory capacity in permeabilized skeletal muscle fibers (*n* = 11–12). (A) with complex I linked substrates (CI_*P*_) (mass‐specific respiratory capacity). (B) Intrinsic mitochondrial respiratory capacity with complex I linked substrates (CI_*P*_) (mass‐specific respiratory capacity normalized to CS activity). (C) with complex I + II linked substrates (CI + II_*P*_) (mass‐specific respiratory capacity). (D) Intrinsic mitochondrial respiratory capacity with complex I + II linked substrates (CI + II_*P*_) (mass‐specific respiratory capacity normalized to CS activity). (E) *K*_m_^app^ for ADP in permeabilized skeletal muscle fibers (*n* = 12). Horizontal lines represent ½*V*_*max*_, and the corresponding vertical lines to the *x*‐axis is apparent *K*_m_ (ADP). The inserted figure illustrates the ADP stimulated respiration and *K*_m_^app^ values on a scale from 0 to 0.5 mmol/L ADP. *Post‐HIT different from pre‐HIT,* P* < 0.05. *Post‐HIT different from pre‐HIT,* P* < 0.05.

CS activity increased (*P* < 0.05), and HAD activity was unchanged following training (Table 2). Skeletal muscle creatine kinase was significantly decreased after the intervention (369 ± 14 to 322 ± 19 U/mg). Mitochondrial respiratory capacity with complex I linked substrates (CI_*P*_) was unchanged after training (Fig. 2A). Maximal respiratory capacity with complex I and II linked substrates (CI + II_*P*_) was significantly increased after training (Fig. 2C), but intrinsic mitochondrial respiratory capacity (i.e., respiratory rates normalized to CS a marker of mitochondrial content) did not change (all substrate combinations) (Fig. 2B and D). Intrinsic mitochondrial respiratory capacity normalized to HAD activity was unchanged following training (data not shown). The substrate control ratio with succinate (SCR, CI_*P*_/CI + II_*P*_) and CI + II_*P*_/CI + II_*E*_ was unchanged, but CI_*L*_/CI_*P*_ control ratio was increased following training (Table 3). Mitochondrial ADP sensitivity decreased after training, with an increased *K*_m_^app^ from 0.14 ± 0.02 to 0.29 ± 0.03 mmol/L (Fig. 2E, *P* = 0.002). Mitochondrial substrate sensitivity for glutamate was unchanged after training (*K*_m_^app^: 0.99 ± 0.17 vs. 0.78 ± 0.09 mmol/L glutamate) as well as *V*_*max*_ (22 ± 2 vs. 22 ± 3 pmol/mg/sec).

## Discussion

The novel finding in this study is that high‐intensity training, which taxes the skeletal muscles, elicits an increase of the intrinsic mitochondrial respiratory capacity in adipose tissue which occurred in the face of a decrease in mitochondrial respiratory capacity (Figure 1A–D). This occurred with no changes in ADP sensitivity (*K*_m_^app^) in the adipose tissue. Furthermore a training induced decreased sensitivity for ADP (increase in *K*_m_^app^) in skeletal muscle mitochondria was found. The decreased ADP sensitivity occurred in the face of an increase in maximal mitochondrial respiratory capacity and mitochondrial content (CS activity) in the skeletal muscle, causing no difference in intrinsic respiratory capacity with the substrates used in this study, although some changes were observed in the calculated ratios (Table 3).

### General adaptations

Following HIT, maximal oxygen uptake increased, which is in accordance with some (Tabata et al. 1996; Ziemann et al. 2011; Shepherd et al. 2013; Larsen et al. 2015) but not all HIT studies (Burgomaster et al. 2008; McKay et al. 2009; Earnest et al. 2013). No changes were observed in fasting glucose and insulin concentrations in this study, which is in line with some studies (Babraj et al. 2009), but not all (Hood et al. 2011).

### Adaptations in adipose tissue

The decreased respiratory capacity was accompanied by a nonsignificant decrease in mtDNA content in the subcutaneous adipose tissue and therefore an increased intrinsic respiratory capacity was found in this study. We note a similar nonsignificant trend as Camera and colleagues did with nearly 30% depression in CS activity in adipose tissue after 10 days of exercise training in humans (Camera et al. 2010). We have previously reported that 6 weeks of high‐intensity training does not affect mitochondrial content or respiratory capacity in SAT (Larsen et al. 2015), the difference between this and the present study was the intensity (128% of maximal load) of the training, and that the gender distribution was different. Altogether, a training induced decrease in adipose tissue mitochondrial contents seems to take place, final proof requires a larger study. We found a reduced ratio between LEAK and mitochondrial respiratory capacity in subcutaneous adipose tissue, after training, which is similar to previous findings in human skeletal muscle after training (Pesta et al. 2011). No difference was seen in mitochondrial respiratory capacity (CI_*P*_) in this study, and it could be speculated that HIT induces a higher degree of coupling of the mitochondria from subcutaneous adipose tissue in this study. No difference was found in ADP sensitivity in adipose tissue following HIT. If the adipose tissue adaptation is derived from the exercising skeletal muscle via exersomes, most likely the adaptation requires more time in the secondary tissue. In this context it is of note that there were no differences in ADP sensitivity between adipose tissue and muscle tissue before training started and that a correlation is seen in ADP sensitivity between adipose tissue and skeletal muscle before the training intervention, but is not present after the intervention.

### Adaptations in skeletal muscle

Mitochondrial respiratory capacity per mg tissue increased in skeletal muscle, in accordance with published data following a combination of endurance, high‐intensity, and strength training (Pesta et al. 2011) and HIT (Jacobs et al. 2013; Larsen et al. 2015). Mitochondrial content (CS activity) was increased after HIT in skeletal muscle, as has previously been reported (Burgomaster et al. 2008; Hood et al. 2011; Larsen et al. 2015). When mitochondrial respiratory capacity was normalized to CS activity in this study, there was no training induced effect on intrinsic mitochondrial respiratory capacity. This illustrates that the improved respiratory capacity per mg tissue was due to increased mitochondrial content, which is in concordance with previous findings (Jacobs et al. 2013; Larsen et al. 2015; Granata et al. 2016) following high‐intensity training. Studies investigating other training intensities have reported changes in intrinsic mitochondrial respiratory capacity, SIT training have resulted in an increased intrinsic capacity (normalized to CS activity) (Granata et al. 2016) whereas regular endurance training resulted in a decreased intrinsic capacity when normalized to mitochondrial volume density (measured by TEM) (Meinild Lundby et al. 2018). In the study by Lundby they also see a numerically decreased intrinsic respiratory capacity when normalized to CS activity although not significant (Meinild Lundby et al. 2018). The differences between the two latter studies could be that they are using different markers for mitochondrial content.

Walsh and colleagues reported a decreased mitochondrial ADP sensitivity following a combination of endurance and high‐intensity training (Walsh et al. 2001a), which is supported by this study. This indicates an inverse relationship between mitochondrial ADP sensitivity and maximal oxygen consumption, which is supported by a study investigating ADP sensitivity in subjects that differ in physical fitness (Zoll et al. 2002). However, we found no correlation between VO_2max_ and ADP sensitivity in this study, and an explanation for the decreased ADP sensitivity with increasing maximal oxygen uptake has not been reported in the literature.

It has been demonstrated that addition of creatine (Cr) to permeabilized muscle fibers ex vivo increases the ADP sensitivity (Walsh et al. 2001b), whereas creatine phosphate (CrP) content has the opposite effect (Walsh et al. 2001b), suggesting that mitochondrial ADP sensitivity is influenced by the Cr‐CrP shuttle system. Both mitochondrial creatine kinase (miCK) and creatine kinase (CK) regulate the Cr‐CrP shuttle system, but only miCK activity increases following endurance training (Apple and Rogers 1986). The miCK only constitutes <10% of total CK activity in trained individuals (Apple and Rogers 1986), and it seems that CK is in excess of available Cr and CrP. Furthermore data from this study also show a reduced CK activity which could indicate that CK is in excess. We speculate that a training induced increase in miCK activity may illicit increased phosphate‐transferring capacity and thereby decrease the ADP sensitivity (Zoll et al. 2002). An increased miCK activity, and possibly miCK sensitivity to Cr, could moreover explain unchanged baseline Cr and CrP levels following sprint or endurance training, as reported previously (Burgomaster et al. 2008).

It has been demonstrated that Adenylate Kinase activity increases with muscle contraction in electrically stimulated rat muscle (Dzeja et al. 1998), but to our knowledge the only study investigating the effect of training on AK activity is by Linossier et al. 1997;. They reported an 18% increase in AK activity following sprint training (two series of 15 intermittent sprints (5 sec sprint and 55 sec rest); 4 times per week for 7 weeks), without any changes in mitochondrial content (CS activity) (Linossier et al. 1997). Given the role of AK in cross membrane phosphate transferring, AK could, at least in part, explain the reduced mitochondrial ADP sensitivity. It has been suggested that AK is primarily coupled to ATP transfer in glycolytic fibers (Dzeja et al. 1998), whereas the main role of CK is to control ADP accumulation (Sahlin and Harris 2011), and primarily in oxidative fibers. In muscle with mixed fibers, as the vastus lateralis, it is likely that an increased activity of both phosphate‐transferring systems increases the mitochondrial efficiency, thus minimizing the need for utilizing added ADP. This could explain the decreased sensitivity observed in this study, although our calculated index of electron coupling efficiency (CI_*L*_ / CI_*P*_) from this study does not support this increased efficiency. However, further studies are required to establish the interaction between CK and AK catalyzed phosphate transferring, and how training influences this interaction. Another explanation for decreased ADP sensitivity following training, could also be the transport of phosphate across the outer (VDAC) and inner mitochondrial membrane (ANT), in combination with miCK as suggested by Perry et al. (2012). It has been reported that 6 weeks of endurance training increases ANT by 100%, but mitochondrial volume was only increased approximately 50% (Fernstrom et al. 2004), indicating that the transporters are increased to a higher extent than mitochondrial volume which potentially could explain the changes in ADP sensitivity. Furthermore, it has been reported that mitochondrial ADP sensitivity differs depending on the muscle fiber type, where oxidative muscle mitochondria in rats (Kuznetsov et al. 1996) and mice (Veksler et al. 1995) have a low sensitivity for ADP and glycolytic muscle mitochondria have a higher ADP sensitivity. We would not expect a major change in fiber‐type distribution after the training period in this study, but we could speculate that the muscle is becoming more oxidative (higher respiratory rates), which could partly explain our finding. Studies have reported changes from more glycolytic type IIX fibers to type IIA after training (Andersen and Aagaard 2000).

## Summary & Perspective

Our results illustrate that 6 weeks of low volume HIT improves the oxidative metabolism in healthy overweight adults, as evidenced by an increased VO_2max_ and CS activity in skeletal muscle. No intrinsic mitochondrial adaptation was seen in skeletal muscle with the given substrates used. In contrast, the oxidative capacity in adipose tissue was decreased following training. We demonstrate that low volume HIT decreases the mitochondrial ADP sensitivity, which could be an indication of a more oxidative muscle. However, future research is necessary to elucidate the mechanisms responsible for alterations in mitochondrial ADP sensitivity.

## Limitations

There are some limitations to this study that needs to be acknowledged. First of all the heterogeneity of the study population is not ideal. Both genders are investigated and the subjects differ in age. We are aware of that this is not ideal, but since we are looking at a training intervention we are investigating individual changes. Another limitation is also the fact, that only one marker of mitochondrial content was used in skeletal muscle (CS activity) and adipose tissue (mtDNA) in this study.

## Conflict of Interest

The authors declare no conflict of interest.
